# An Assessment of the Population of Cotton-Top Tamarins (*Saguinus oedipus*) and Their Habitat in Colombia

**DOI:** 10.1371/journal.pone.0168324

**Published:** 2016-12-28

**Authors:** Anne Savage, Len Thomas, Katie L. Feilen, Darren Kidney, Luis H. Soto, Mackenzie Pearson, Felix S. Medina, German Emeris, Rosamira R. Guillen

**Affiliations:** 1 Conservation Department, Disney’s Animals, Science and Environment, Lake Buena Vista, FL United States of America; 2 Centre for Research into Ecological and Environmental Modelling and School of Mathematics and Statistics, University of St. Andrews, St. Andrews, Scotland, United Kingdom; 3 Fundación Proyecto Tití, Barranquilla, Colombia; Università degli Studi di Napoli Federico II, ITALY

## Abstract

Numerous animals have declining populations due to habitat loss, illegal wildlife trade, and climate change. The cotton-top tamarin (*Saguinus oedipus*) is a Critically Endangered primate species, endemic to northwest Colombia, threatened by deforestation and illegal trade. In order to assess the current state of this species, we analyzed changes in the population of cotton-top tamarins and its habitat from 2005 to 2012. We used a tailor-made “lure strip transect” method to survey 43 accessible forest parcels that represent 30% of the species’ range. Estimated population size in the surveyed region was approximately 2,050 in 2005 and 1,900 in 2012, with a coefficient of variation of approximately 10%. The estimated population change between surveys was -7% (a decline of approximately 1.3% per year) suggesting a relatively stable population. If densities of inaccessible forest parcels are similar to those of surveyed samples, the estimated population of cotton-top tamarins in the wild in 2012 was 6,946 individuals. We also recorded little change in the amount of suitable habitat for cotton-top tamarins between sample periods: in 2005, 18% of surveyed forest was preferred habitat for cotton-top tamarins, while in 2012, 17% percent was preferred. We attribute the relatively stable population of this Critically Endangered species to increased conservation efforts of Proyecto Tití, conservation NGOs, and the Colombian government. Due to continued threats to cotton-top tamarins and their habitat such as agriculture and urban expansion, ongoing conservation efforts are needed to ensure the long-term survival of cotton-top tamarins in Colombia.

## Introduction

Many species and ecosystems are currently threatened due to anthropogenic pressures such as habitat loss, illegal hunting, and global climate change. Evaluating the success of efforts to preserve biodiversity is of urgent global concern. The Convention on Biological Diversity, first realized in 1994, proposed achieving a significant reduction in the rate of biodiversity loss globally by 2010 [1]. However, an evaluation of 31 cross-disciplinary indicators that measured the biodiversity status, the anthropogenic pressures (i.e., deforestation), and conservation strategies (i.e., the number of protected areas) on a global level from 1970 to 2010 demonstrated that such reduction has not been achieved [2]. While most of the indicators demonstrated losses of biodiversity, this assessment found small scale successes such as increases in the number of protected areas, down-listing of many species on the IUCN Red List, and increases in funding to assist in biodiversity conservation [2, 3]. Some studies have demonstrated that species recovery is possible [2, 4–7], and nearly 7% of mammals, birds, and amphibians classified as Threatened or Near Threatened on the IUCN Red List [5] have stable or increasing populations mostly due to conservation actions that protect habitats and reduce human-wildlife conflict over resources [3].

The cotton-top tamarin (*Saguinus oedipus*) is a small arboreal primate endemic to northwestern Colombia that is currently threatened with extinction due to habitat loss and capture for the illegal pet trade [8]. They live in family groups of 5–12 individuals that defend territories of 2–12 ha [9]. Cotton-top tamarins were likely to have been abundant in Colombia, as 20–30,000 individuals were captured in the late 1960’s for use in biomedical research [10], but in 1973 the species was declared Endangered by IUCN. A population survey conducted in 2005 estimated less than 7,500 individuals remaining in the wild [11]. This estimate, combined with the dramatic loss of suitable forest habitat [12] led to the reclassification of the cotton-top tamarins as Critically Endangered [13] and placement on the list of the World’s 25 Most Endangered Primates 2008–2010 [14].

With an increased interest in conservation from both governmental authorities and conservation organizations, forest recovery trends in Colombia appear promising [15, 16]. Colombia had an estimated increase of 16,963 km^2^ in net woody vegetation between 2001 and 2010 [17], and protected areas in Colombia effectively reduced the probability of forest clearing from 2002 to 2005 [17]. Despite increases in forested and protected areas, forest conversion for agriculture use, cattle ranching, and palm oil plantations still happens on privately owned lands within the historic distribution of cotton-top tamarins [18]. As the total human population in Colombia increased from 16 to 47 million people between 1960 and 2012 [19], and with more than 27.8% of the population living in poverty as of 2015 [20], human impacts on biodiversity represents a serious threat to biodiversity conservation in Colombia.

Although forest coverage is improving, pressures such as agriculture and urban expansion continue to threaten the habitat of cotton-top tamarins. In order to monitor the status of the population of cotton-top tamarins, we aimed to resurvey the remaining wild population of cotton-top tamarins and their habitat in 2012. We compared these values to an initial survey that took place in 2005 to examine how the population of cotton-top tamarins and their habitat changed over time.

## Material and Methods

### Survey Design

We replicated the survey design and procedures described in [11] to estimate cotton-top tamarins’ population in 2012 and to compare it with results from the 2005 survey. Using remote-sensed spectral data covering the historic distribution of cotton-top tamarins in northwestern Colombia in 2000, we identified 14,534 km^2^ of tropical dry forest patches. At the time of the 2005 survey, 10,515 km^2^ of this land was inaccessible due to political conflict in the area. Of the 4,019 km^2^ that was safely accessible to the field team, much of the forested area previously identified in the satellite images was already deforested, misidentified, or smaller than necessary to sustain a tamarin population (less than 0.005 km^2^), leaving only 98.93 km^2^ of tropical dry forest in 43 forest patches available to be surveyed. Of the 43 identified patches, our field team was able to access 36 patches in 2012. Patch size ranged from 0.18 to 17.35 km^2^ (mean forest patch size = 2.3, SD = 3.49 km^2^). To survey both the monkeys and forest quality, we placed transects that were 1.5 km long, 200 m wide, with the long axis oriented north-south, and using a systematic random design with a between-transect spacing of 400m. The number of transects per forest patch varied between 3 and 51 (mean number of transects per patch = 8.5, SD = 9.4) depending on the size of the patch and how the systematic random transects intersected the forest. The study design marked 303 transects to be surveyed; due to political conflict or lack of permission from the landowners, our field team was able to survey 247 transects in 2005 and 235 transects in 2012 (208 of these were surveyed in both 2005 and 2012).

### Field Methods

The field team surveyed areas using a “lure strip transect” method [11] from January through June 2012. Monkeys were surveyed in the morning, between 0700–1100, as this is the period of the day they are most active. Two teams walked parallel to each other 200 m apart, with both teams playing recordings of unknown cotton-top tamarins long calls (continuous play of 60 s of calls at 80 dB followed by 30 s of silence) from a high-quality playback device. The broadcast loudspeakers were generally oriented to cover forest ahead of each team as they proceeded along the transect. Previous validation trials of the method, using the long-term field site of Proyecto Tití, showed that: (a) all groups responded from within the strip of 200m width formed by the two teams; (b) it was possible to distinguish between those responding from inside and outside the strip; and (c) groups became rapidly habituated and stopped responding after multiple trials [11]. In the 2012 survey, as in the 2005 survey, the team surveyed each transect once to avoid habituation.

When cotton-top tamarins were observed, the field team recorded the compass bearing the group arrived along, the location of the animals along the transect and the number of adults, juveniles, and infants.

Vegetation type was classified along the transect every 50 m as (a) primary forest, (b) secondary forest, (c) forest with cleared undergrowth, (d) area of regrowth with young trees producing fruits, (e) area of regrowth without fruiting trees, (f) open pasture or agriculture land, or (g) land converted for human settlements. Based on previous behavioral research [9], we attributed a preference index to each habitat type (a-g) reflecting its use by the tamarins: 0 (non-suitable habitats: e, f, g), 1 (habitats used only for feeding: d) and 2 (preferred habitats: a, b, c).

### Statistical analyses

R Statistical Software [21] was used for all statistical analyses. We calculated the cotton-top tamarin population size by using the following formula,

$$\hat{N}=\frac{n_{s}\hat{s}A}{a}$$

where $\hat{N}$ is the total estimated population size, *n*_*s*_ is the number of groups encounters, *ŝ* is the estimated mean group size, *A* is the total area of the study region, and *a* is the area sampled by the transects. We calculated population size estimates for each forest patch, and combined these estimates to calculate totals for each department and for the entire population. As each department has an autonomous environmental authority that dictates environmental policy and how protected areas are managed, it was important to analyze the data at the level of the department. As the density of animals detected in the core forest patch was greater than the edge of the forest, we calculated encounter rates and variance for the core forest area (the mapped patch plus 30 m margin) and the buffer area (area 300 m border around core forest area) separately.

We used an improved estimator of encounter rate variance that accounts for the systematic nature of the survey design (i.e., with transects laid out on a systematic random grid). The previous estimator (Eq 4 of reference 11)

$$\hat{var}\left(\frac{n_{s}}{a}\right)=\frac{1}{a(k-1)}\sum_{i=1}^{k}a_{i}{\left(\frac{n_{s,i}}{a_{i}}-\frac{n_{s}}{a}\right)}^{2}$$

assumed a completely random layout of transects, and tends to over-estimate variance given a systematic design [22]. Hence, we instead used the estimator for systematic designs (Eq 16 of [22]–estimator O2)

$${\hat{var}}_{O2}\left(\frac{n_{s}}{a}\right)=\frac{2k}{a^{2}(k-1)}\sum_{i=1}^{k-1}\frac{{\left(a_{i}a_{i+1}\right)}^{2}}{{\left(a+a_{i+1}\right)}^{2}}{\left(\frac{n_{s,i}}{a_{i}}-\frac{n_{s,i+1}}{a_{i+1}}\right)}^{2}.$$

This involves pairing up adjacent transects into overlapping pseudo-strata; in our study we used all pairs of adjacent transects to form pairs. In order to compare the 2005 survey results to those of 2012, we reanalyzed the 2005 data using the improved estimate of encounter rate variance.

We used the number of groups rather than the number of individuals sighted to calculate the encounter rate because it resulted in a lower variation between transects within patches. In order to determine the appropriate geographic level to estimate mean group size, we performed a model selection exercise. We used the observed group size as the response variable, and location within the forest patch (buffer or core), forest patch identity, and department as factor covariates. We then ran a zero-truncated Poisson regression. The model with the lowest Akaike Information Criterion [23] was used to determine group size.

We used the delta method to calculate variance on population size N given ${\hat{var}}_{O2}(n_{s}/a)$ and $\hat{var}(s)$, and we calculated 95% confidence intervals by assuming N was a log-normal random variable [24].

To compare changes in forest suitability for tamarins between 2005 and 2012, we calculated the frequency of each category of habitat suitability criteria (preference index from 0 to 2) for each department and across the surveyed area and compared it using a Chi-Squared Test of Independence. We also compared changes in forest coverage of each segment between 2005 and 2012 to determine any significant deforestation or regrowth events.

### Ethics Statement

We obtained approval for this study from the Disney’s Animal Care and Use Committee and permission to conduct research in Colombia as part of an institutional agreement with Fundacíon Proyecto Tití and CARDIQUE, the local environmental authority in Colombia. The study accessed both privately owned and protected areas in Colombia. For areas conducted on private lands, we obtained permission from the landowners, and for public lands, we obtained permission from the Corporaciones Autonomas Regionales de Colombia, CRA, CARDIQUE, CORPOURABA, CVS, CARSUCRE, CORANTIOQUIA, CORPOMOJANA, and Parques Nacionales Naturales de Colombia (No. 00106–816–0012507). This study involved wild non-human primates in their natural environment. Researchers did not have any direct physical contact with the monkeys.

## Results

During the 2012 survey, 39.39 km^2^ were surveyed in 36 forest patches. Cotton-top tamarins were recorded in 29 of 36 surveyed patches, totaling 816 individuals in 75 group encounters, with an average encounter rate of 1.7, SD = 1.8 groups per km^2^. Of the 816 tamarins, 89% were adults, 3.6% were juveniles (less than 1-year-old), and 7.2% were infants (less than 3 months).

Groups consisted of 2 to 10 individuals, with the average group size being 4.6, SD = 1.8 individuals. The AIC-best model of group size had core vs. buffer as the explanatory variable (Table 1). This model was considerably better (in AIC terms) than the next best model: we calculated AIC weights [23] (also called model weights) for each model and the core vs. buffer model had 95% of the total weight (Table 1). We, therefore, used the core vs. buffer model for inference. Note, that none of the models explained much of the variation in group size (percentage deviance explained <16%, Table 1), indicating that other factors than those we were able to model must play a larger part in determining group size.

**Table 1:** Model fit and model selection results for zero–truncated Poisson regression models fit to group size data.

Factor covariate	Number of Parameters	% deviance explained	Akaike Information Criterion, AIC	ΔAIC	AIC weight
Core/buffer	2	5.56	681.37	0	0.95
None	1	0	687.28	5.92	0.05
Department	5	1.37	693.33	11.96	0.00
Forest patch	27	15.33	717.45	36.08	0.00

We estimated the population size in each patch (Table 2) totaling 1,919 cotton-top tamarins (95% CI: 1,622–2,270; CV: 9%) in all surveyed area, which covers 27% of the species range. Extrapolating our density estimates to the un-surveyed areas within species range, the estimated population of tamarins in the wild in 2012 was 6,946 individuals.

**Table 2:** Forest patches, cotton-top sightings, and estimated population size of surveyed areas in 2012.

Department	Patch Name	UnbufferedArea (km^2^)	Buffered Area (km^2^)	K	Area Sur–veyed (km^2^)	Ns	% CV	DF	Mean Estimate	LCL	UCL
Antioquia	Buenos Aires	2.47	5.50	13	1.38	3	40	111	55	25	118
Antioquia	Contadora 1	0.85	2.56	6	0.71	0	0	0	0	0	0
Antioquia	Contadora 2	0.72	2.27	6	0.67	0	0	0	0	0	0
Antioquia	Cucharal	0.83	2.66	9	0.79	2	54	60	22	8	60
Antioquia	El Muerto	0.66	2.22	6	0.58	0	0	0	0	0	0
Antioquia	El Olivido	1.30	3.06	10	0.81	1	64	59	18	6	58
Antioquia	La Pampa	0.95	1.92	6	0.48	3	37	60	56	28	116
Antioquia	La Pradera	1.54	3.82	10	1.01	5	31	117	78	43	143
Antioquia	Las Cruces	1.01	2.70	6	0.75	2	52	86	36	14	95
Antioquia	Leticia 1	1.34	3.49	7	0.67	1	86	59	21	5	94
Antioquia	Leticia 2	0.38	1.67	5	0.47	2	51	113	30	12	79
Antioquia	Marimonda	17.35	51.08	51	6.28	9	26	117	305	182	508
Antioquia	Mellito	3.53	6.62	14	1.88	5	29	61	103	58	183
Atlantico	Luruaco	8.95	14.54	18	1.87	3	46	102	109	46	258
Atlantico	Pioho	0.37	2.04	4	0.47	1	84	59	17	4	73
Bolivar	Arroyo De Pierda	0.44	2.01	6	0.39	0	0	0	0	0	0
Bolivar	Bayunca	1.78	4.04	0	0	0	67	118	29	9	97
Bolivar	Ceibal	3.87	8.02	16	19.6	5	33	115	92	49	173
Bolivar	Colorado	0.18	1.11	0	0	0	140	118	7	1	57
Bolivar	Hac. Sta Catalina	1.06	3.31	0	0	0	77	118	23	6	88
Bolivar	San Juan Nepo	14.20	21.22	37	4.90	7	26	118	143	85	239
Cordoba	Aguas Prietas	5.49	9.56	18	1.71	2	48	60	58	23	143
Cordoba	Chimborazo 1	0.56	1.91	5	0.43	0	0	0	0	0	0
Cordoba	Chimborazo 2	0.42	1.68	5	0.38	0	0	0	0	0	0
Cordoba	El Bosque	0.79	2.89	3	0.08	0	85	59	16	4	69
Cordoba	El Campano	1.26	3.50	10	0.86	2	44	60	31	13	71
Cordoba	El Turro	1.13	2.63	8	0.79	1	63	59	17	5	54
Cordoba	Esmeralda	1.26	2.97	7	0.75	4	37	119	70	35	142
Cordoba	Galicia	1.36	3.42	10	0.90	3	37	60	55	27	113
Cordoba	Griton	0.64	1.68	5	0.38	2	46	60	43	18	104
Cordoba	La Ceiba	1.32	3.64	0	0	0	64	75	29	9	93
Cordoba	La Gloria	0.94	2.36	3	0.18	0	81	59	17	4	72
Cordoba	La Habana	0.78	2.35	6	0.60	0	0	0	0	0	0
Cordoba	Zamorano	0.41	1.67	5	0.46	0	0	0	0	0	0
Sucre	Buenos Aires	1.01	3.00	0	0	0	67	93	28	8	93
Sucre	El Salado	2.22	4.63	0	0	0	49	94	51	21	128
Sucre	Las Navas	5.37	9.12	19	1.92	7	28	121	151	88	258
Sucre	Ojo de Agua	1.35	3.58	10	0.98	0	0	0	0	0	0
Sucre	Rabon	5.08	8.80	0	0	0	33	95	108	57	206
Sucre	Santa Ines	0.41	1.84	6	0.42	0	0	0	0	0	0
Sucre	Tigua	0.97	2.73	6	0.82	0	0	0	0	0	0
Sucre	Varsovia	2.05	4.96	14	1.23	5	29	61	101	57	179
Sucre	Villa de Helena	0.31	1.65	5	0.43	0	0	0	0	0	0
**Total**		**98.93**	**226.44**	**375**	**39.39**	**75**	**9**		**1,919**	**1,622**	**2,270**

K = Number of transects, Ns = Number of observed groups, % CV = Percent coefficient of variation on this estimate, DF = Degrees of freedom, LCL = Lower confidence limit, UCL = Upper confidence limit

**Fig 1:**
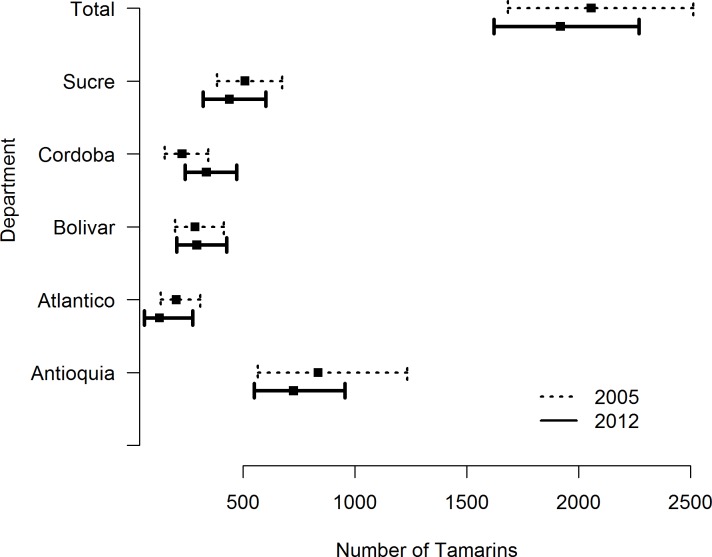
Estimated population size (with 95% confidence interval) of cotton-top tamarins in surveyed areas in Colombia (overall and separated by department) and in each department in 2005 and 2012.

There was little change in the population estimates from 2005 to 2012 within the sampled areas (Fig 1). Recalculating the 2005 data with the same method as 2012 data, we estimated the population to be 2,056 individuals (95% CI: 1,683–2,513; CV: 10%). The point estimate is slightly different from that reported in [11] (of 2,045) due to a small change in the way we accounted for transects that span multiple forest patches. As expected, the variance is lower (the previous CV was 13%) because of the improved encounter rate variance estimator.

Extrapolating the 2005 density estimate to the species’ range, we estimate there were 7,443 animals in 2005. The estimated population change between surveys period was –7%, which represents a decline of approximately 1.3% per year.

**Fig 2:**
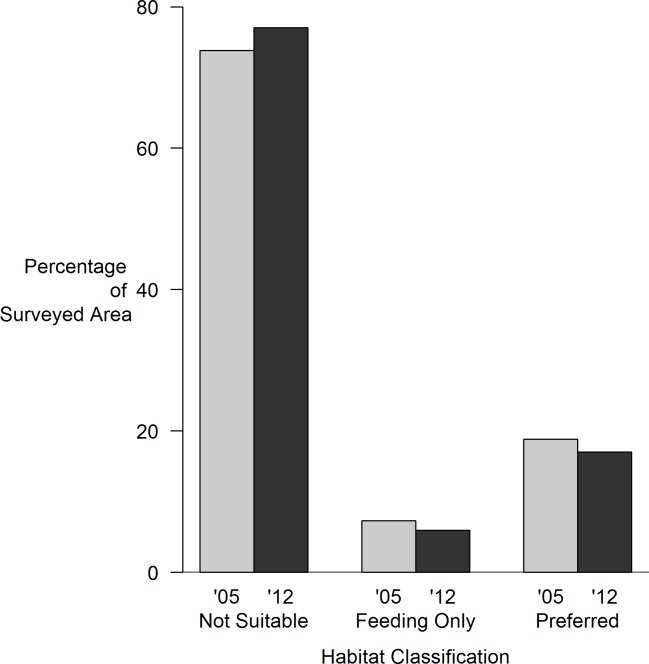
Changes in suitable forest for cotton-top tamarins from 2005 to 2012.

Of the 11,794 segments surveyed in 2005, 73.8% were not suitable, 7.3% were suitable for feeding, and 18.8% were preferred. In 2012, 77.0% were not suitable for cotton-top tamarins, 6.0% were suitable only for feeding, and 16.7% were preferred tamarin habitat: suggesting no significant differences in habitat suitability from 2012 (Fig 2; χ = 3.1, df = 3, p = 0.37).

We found similar trends by departments since neither Cordoba (χ = 0.12, df = 1, p = 0.72), Sucre (χ = 0.11, df = 1, p = 0.73), Antioquia (χ = 0.04, df = 1, p = 0.82), Atlántico (χ = 0.30, df = 1, p = 0.58), or Bolivar (χ = 0.003, df = 1, p = 0.95) experienced any significant changes in suitability of habitat for cotton-top tamarins from 2005 to 2012.

## Discussion

The results of our survey demonstrate that the population of cotton-top tamarins has remained relatively stable from 2005 to 2012, at least in the regions we were able to survey. Additionally, there has been very little change in the available habitat for cotton-top tamarins during this time period. Although the total number of animals remaining in the wild is fairly low, a stable population for a Critically Endangered species can be considered a conservation success. Additionally, using the methods of Fewster [22], we were able to improve our population estimates by capitalizing on the systematic random design used to produce a lower estimated variance.

Population estimates give a general understanding of a species status. However, they can often obscure underlying population dynamics such as compression effects (higher densities of animals due to less available habitat) [25] or source-sink dynamics (high-quality habitat with population growth supporting poor quality areas with population declines) [26]. With our current dataset, it is difficult to determine what population dynamics contributed to the relatively stable population size from 2005 to 2012. Although we cannot determine if some of these surveyed populations are potential ecological sinks, additional data from other studies on this species suggest that stability is an accurate representation of the cotton-top tamarin population for the sampled areas [27, 28]. Reproductive rates of cotton-top tamarins from a long-term research project at Hacienda Ceibal (Bolivar), one of the surveyed patches, have remained stable: on average each adult female produces two offspring a year [27] and infant mortality rates are low [28].

Stability of the population can be attributed to decreases in threats. We found that forest suitability for tamarins has not changed from 2005 to 2012. At the level of individual parcels, some forests experienced slight deforestation, while others experienced regrowth. Although the majority of sample was considerably impacted by agricultural expansion and therefore unsuitable territory for tamarins, most parcels consisted of a mosaic landscape, with small sections of secondary forest (preferred habitat for tamarins) near the center of forest patches, areas of regrowth, agricultural lands used and ranching usually located closer to human settlements.

One proposed explanation for the stability of the cotton-top tamarins and habitat is the work of conservation organizations. Since 1987, Proyecto Tití has been leading the effort to bring increased national and international attention to the conservation efforts of cotton-top tamarins in Colombia [29] through scientific investigation [9, 28, 30], community empowerment and sustainable development [31, 32], education [8], training of Colombian nationals, and protection of habitat in the department of Atlántico and Bolívar [8]. Proyecto Tití directly lead in the protection and conservation of habitat by working to establish two protected areas: El Parque Natural Regional Bosque Seco El Ceibal Mono Tití (Cotton-top Tamarin Regional Park in Bolívar) [33] and Parque Natural Regional Los Rosales in Atlántico [34], which added legal protection to 1,724 hectares of forest. With their educational programs and public awareness campaigns, Proyecto Tití encouraged local people to help blocked the development of an international airport in the department of Atlántico, which would have decimated two large forest parcels used by cotton-top tamarins that contain more than 200 individuals [35].

The actions of Proyecto Tití to preserve habitat is just one example of a larger trend in Colombia to increase protected areas. Colombia has 627 protected areas covering 23% of its land [36], 214 of these areas have been created since 2005, the year of the first survey. ProAves and World Land Trust established 21 reserves for the protection of birds and other wildlife, including one dedicated to the cotton-top tamarin (*Reserva Natural Tití Cabeciblanco*; [37, 38], while Conservation International Colombia and MPX are conserving 1,100 hectares of tropical dry forest in northern Colombia. These actions reduce the risk of losing key habitat for cotton-top tamarins and other species that use tropical dry forests in Colombia.

Although not directly measured in this study, another possible contributing factor to the relatively stable population is the reduction of animals removed from the wild. In 1973, the Convention on International Trade in Endangered Species of Wild Fauna and Flora (CITES) was written [39]. As cotton-top tamarins are an Appendix 1 species (species that threatened with extinction and have the highest level of protection under CITES), this made any trade of cotton-top tamarins illegal, and reduced the export for biological research. Also, the awareness and education programs of organizations such as Proyecto Tití has decreased the desire to have wild animals as pets in Colombia. In a household survey, results showed that in areas where Proyecto Tití either had economic development programs or education programs, no cotton-top tamarins or other mammals were reported as pets [8].

Large scale development projects, such as mining operations [40], palm oil plantations [41], and dams [42], have the potential to destroy large areas of habitat. As most of the remaining habitats for cotton-top tamarins are small isolated parcels of forest, it is important to focus efforts on providing protection for remaining land areas, connecting small forest fragments to larger protected areas, and increasing the connectivity of forest fragments to allow for population growth and genetic exchange. Efforts that engage local farmers that want to work on their own protected areas have the potential to increase habitat for cotton-top tamarins.

Both the cotton-top tamarin population and the available habitat had few changes from 2005 to 2012. Although we were not able to measure directly the causes of this stability, we propose that long-term, multi-disciplinary conservation programs may have contributed to this result. Through the efforts of Proyecto Tití and the organizations involved in the development of The National Plan for Cotton-top Tamarins, there is now a concerted effort to protect cotton-top tamarins in Colombia. We encourage scientists and conservation managers to think critically about the threats to their species and develop integrated strategic programs, especially at the grassroots level, that can help to reduce the impacts of threats. Careful surveying of critically endangered species using replicable survey methods is an essential action to monitor the status of threatened animals and measure the potential impact of conservation interventions.

## Supporting Information

S1 Table Survey Data for Cotton-top Tamarins in 2012 (XLSX)

S2 Table Survey Data for Cotton-top Tamarins in 2005 (XLSX)

S3 Table Habitat Assessment Data from 2005 and 2012 (XLSX)
